# Antibiotics guided by metagenomic next-generation sequencing to control infection after total knee arthroplasty: A case report and literature review

**DOI:** 10.1097/MD.0000000000046734

**Published:** 2025-12-19

**Authors:** Hao Mei, Jie Mei, Yunxin Sun, Qiang Shang, Peilei Sun, Yubao Yang, Jinqing Kan, Xiaobing Chen, Luchun Sun

**Affiliations:** a Department of Orthopedics, Linyi Maternal and Child Health Hospital (Graduate School Campus), Linyi City, Shandong Province, China; b Department of Rehabilitation Medicine, Linyi Maternal and Child Health Hospital (Graduate School Campus), Linyi City, Shandong Province, China; c Department of Surgery, Shandong Medical College, Linyi City, Shandong Province, China.

**Keywords:** bacterial culture, *C. burnetii*, metagenomic next-generation sequencing, Periprosthetic joint infection, total knee arthroplasty

## Abstract

**Rationale:**

A prosthetic joint infection is a serious complication of joint surgery, with Staphylococcus aureus being the most common pathogen. In contrast, *C. burnetii*, the agent of Q fever, is a rare zoonotic parasite primarily found in cattle and sheep. It can be transmitted through respiratory, digestive, and cutaneous routes, destroying host cells and leading to diseases such as bone and joint infections, endocarditis, and interstitial lung disease.

**Patient concerns:**

A 75-year-old male patient underwent total knee arthroplasty due to degenerative disease in his left knee. After surgery, he was exposed to cattle and their feces. Fifteen months after the operation, he experienced pain, though there was no significant elevation of erythrocyte sedimentation rate (ESR), C-reactive protein (CRP) and white blood cell (WBC). Oral nonsteroidal anti-inflammatory drugs (NSAIDs) were administered. The pain intensified and was accompanied by swelling. ESR and CRP were elevated, while WBC remained normal. The patient took oral Rifampicin for 28 days without improvement. The knee joint puncture culture was negative. The metagenomic next-generation sequencing (mNGS) detected *C. burnetii*, and oral Doxycycline for 1 week. The intravenous infusion of Doxycycline and Moxifloxacin continued for 28 days. After the above indexes stabilized, a 1-stage revision surgery was performed, and Doxycycline and Moxifloxacin were administered for 16 days until the indexes returned to normal and symptoms such as knee joint pain and swelling disappeared.

**Diagnoses:**

Left knee radiography, laboratory tests, and knee cavity puncture, culture, and mNGS testing were performed to confirm the diagnosis of the pathogen.

**Interventions:**

According to mNGS, the left knee was revised and antibiotics were applied before and after the operation until the infection indexes returned to normal and symptoms such as knee pain and swelling disappeared.

**Outcomes:**

ESR, CRP, and WBC indexes were normal. Pain and swelling disappeared. Other symptoms disappeared. Joint flexion and extension mobility was good.

**Lessons:**

In patients with postoperative prosthetic joint infection after total knee arthroplasty, mNGS can identify pathogenic bacteria, inform the use of antibiotics, and enable prompt surgical intervention.

## 1. Introduction

A prosthetic joint infection (PJI) is an infection of a joint prosthesis and the surrounding soft tissues. PJI is a serious complication with an incidence rate of approximately 1% to 2%.^[1–4]^ The diagnosis of PJI is based on clinical presentation, laboratory parameters, and synovial fluid analysis. The treatment of PJI depends on whether the infection is acute (occurring within 6 weeks of the initial surgery) or chronic (occurring more than 6 weeks after the initial surgery). The standard of care for chronic PJI involves removing the implant, thoroughly irrigating and debriding the area, placing an antibiotic spacer, administering prolonged intravenous antibiotic therapy against cultured pathogens, and performing total joint arthroplasty once the infection has resolved.^[5]^ Chronic PJI requires this type of treatment because, once a biofilm forms on the implant, antibiotics alone cannot eradicate the infection.^[5]^ Despite standard treatment regimens, the morbidity and mortality rates associated with PJI remain high. Complications include poor treatment outcomes, disability, persistent infection, the need for amputation, sepsis, and death.^[6]^ The 1-year mortality rate for PJI is 5% to 10%, and the 5-year mortality rate approaches 25%.^[6]^

Staphylococcus aureus is the most common pathogen that causes infection in artificial joints.^[1]^ In this study, we present a case study of a 75-year-old male with chronic PJI caused by the rare pathogen *C. burnetii*, which occurred 1.5 years after total left knee arthroplasty surgery. *C. burnetii* is a specialized intracellular, gram-negative, microscopic bacterium of the genus Coxiella that is widely distributed in nature. This important class of intracellular parasitic organisms of zoonotic origin is mainly transmitted through inhalation of the pathogen via the respiratory tract. Farmers, veterinarians, abattoir workers, and laboratory workers are susceptible to infection, which may lead to unspecified fever, commonly known as Q fever, as well as pneumonia, cardiac arrest, heart attack, and other conditions, including fever, pneumonia, endocarditis, hepatitis, and myelitis.^[7]^ However, it has rarely been reported as the causative agent of artificial joint infections. This study describes the clinical manifestations, diagnosis, and treatment of PJIs caused by *C. burnetii* and highlights some rare etiologies of PJIs that are under documented in the literature.

## 2. Case presentation

**Figure 1.**
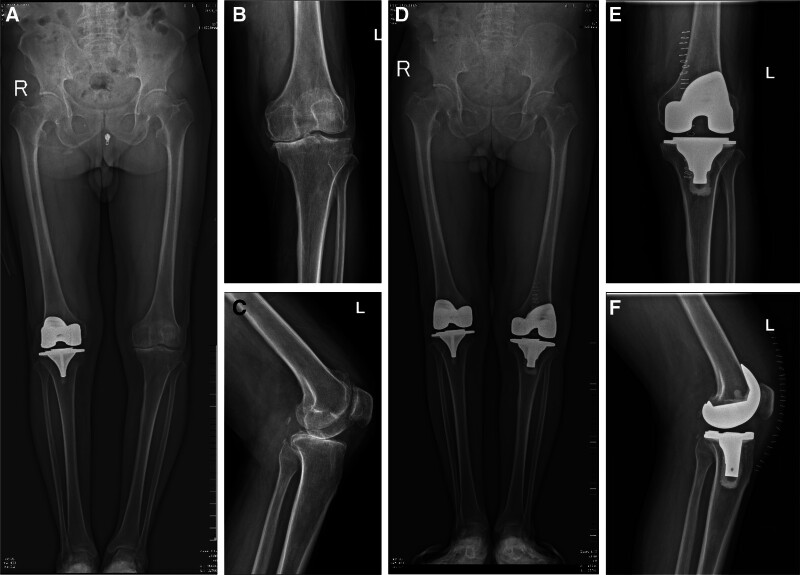
(A–F) radiographic progression of the left knee: (A) Preoperative full-length bilateral lower extremity radiograph; (B) preoperative anteroposterior projection radiograph demonstrating severe osteoarthritis; (C) preoperative lateral projection radiograph; (D) postoperative full-length bilateral lower extremity radiograph; (E) postoperative anteroposterior projection radiograph demonstrating the artificial prosthesis was in a good position without loosening or fracture; (F) postoperative lateral projection radiograph.

**Figure 2.**
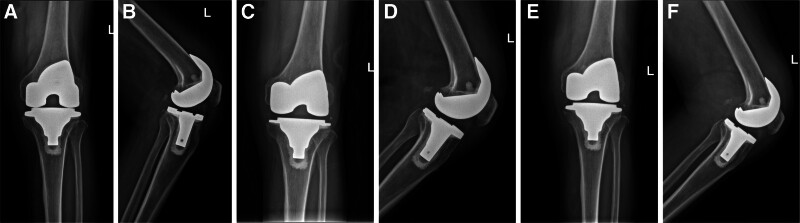
(A–F) radiographic progression of the left knee: (A) postoperative 37 d anteroposterior projection radiograph; (B) Postoperative 37 d lateral projection radiograph; (C) Postoperative 9 mo anteroposterior projection radiograph; (D) postoperative 9 mo lateral projection radiograph; (E) postoperative 16 mo anteroposterior projection radiograph; (F) postoperative 16 mo lateral projection radiograph.

**Figure 3.**
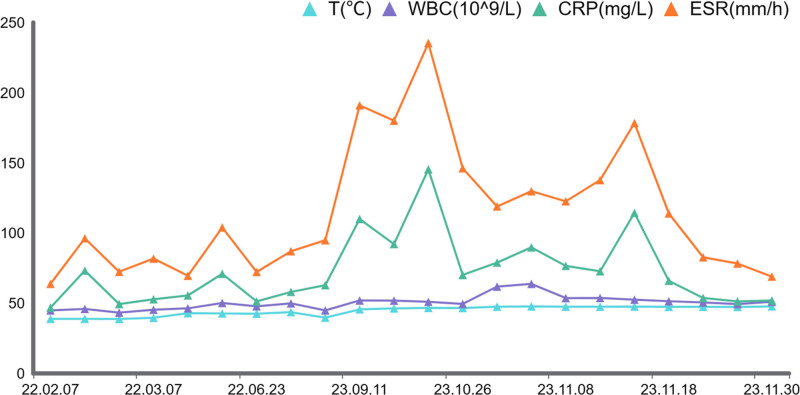
Relationship between the change of inflammatory markers (WBC, ESR, and CRP). CRP = C-reactive protein, ESR = erythrocyte sedimentation rate, WBC = white blood cells.

**Figure 4.**
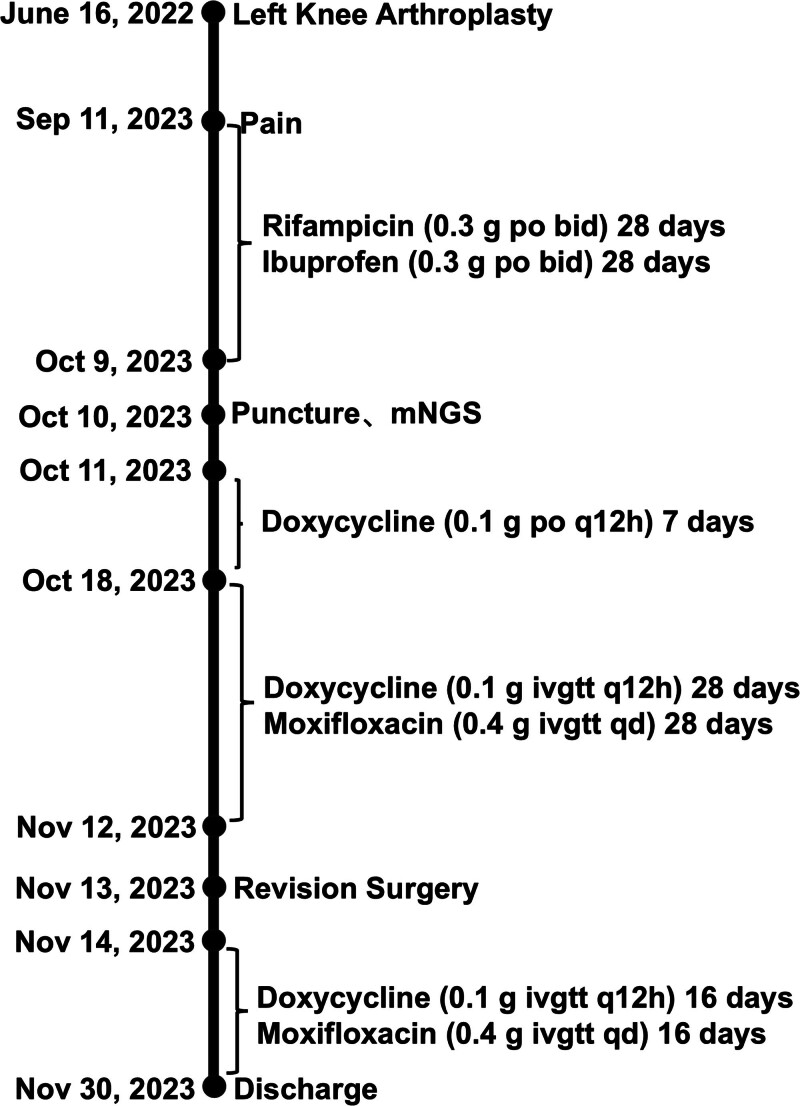
Treatment timeline.

**Figure 5.**
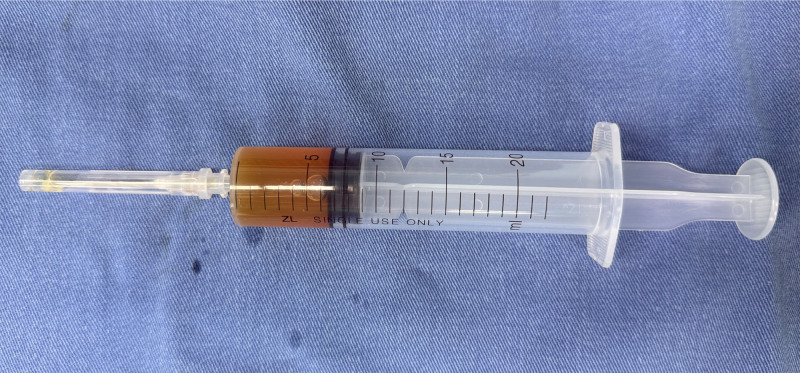
Joint fluid slightly turbid, golden yellow in color, clots, positive Levantine test (3 + white thick cloudy), total number of nucleated cells 550 × 10^6^/L, percentage of single nucleated cells 20%, percentage of multiple nucleated cells 80%, erythrocyte count 12,000 × 10^6^/L.

**Figure 6.**
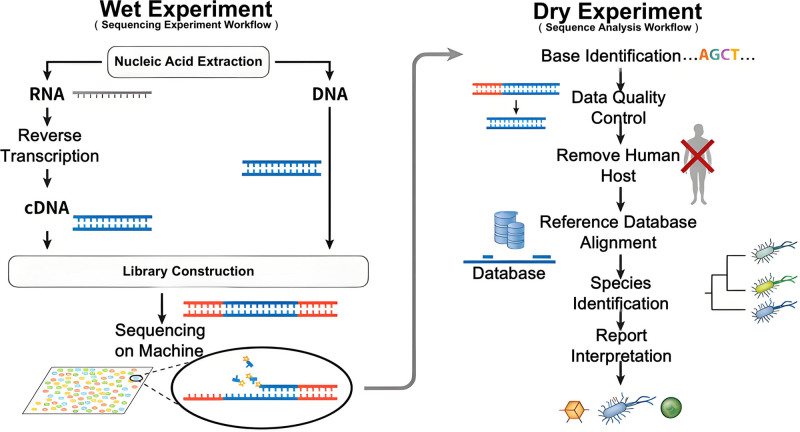
mNGS testing process. mNGS = metagenomic next-generation sequencing.

A 75-year-old Chinese male patient who has long resided in a rural area, with no history of underlying conditions such as diabetes, hypertension or immunodeficiency, underwent his first total left knee arthroplasty 20 months after undergoing total right knee arthroplasty due to degenerative changes in the left knee (Fig. 1A–F). The left knee radiography was reviewed 37 days after surgery, showing that the prosthesis was in good position with no loosening or signs of infection (Fig. 2A and B). Fifteen months after surgery, he began to experience mild swelling of the left knee. The skin temperature was normal, and the joint had good mobility. A radiography (Fig. 2C and D) showed that the prosthesis was in a good position, with no signs of loosening or infection. Laboratory tests showed that his erythrocyte sedimentation rate (ESR) was 32 mm/h, his CRP level was 18 mg/L, and his white blood cell (WBC) count was 5.15 × 10⁹/L (Fig. 3). Symptomatic supportive treatment was administered to reduce swelling and relieve pain. Sixteen months later, the pain and swelling in the left knee joint increased. The symptoms worsened, and the patient was evaluated again in the outpatient clinic. Upon examination, the knee joint was swollen with high skin temperature and compression and movement pain. A radiography of the left knee was performed (Fig. 2E and F). The prosthesis was well-positioned and did not appear loose. Samples were collected for laboratory tests that showed an ESR of 81 mm/h, a CRP level of 58 mg/L, and a WBC count of 5.15 × 10⁹/L (Fig. 3). The WBC count was 6.38 × 10⁹/L. The patient was given Rifampicin (0.3 g po bid) Ibuprofen (0.3 g po bid) as symptomatic supportive treatment in the outpatient clinic for 28 days (Fig. 4). There was no significant improvement in symptoms, so the patient visited the clinic again and underwent knee arthrocentesis (Fig. 5). The knee joint effusion was tested for bacterial culture and mNGS (Fig. 6). The results of the bacterial culture were negative, whereas the 24-hour mNGS test returned a result of *C. burnetii*. How was the patient infected with this bacterium? A careful review of the patient’s history revealed that he was discharged from the hospital to clean the herd and barn for 1 day. Combined with these results and a history of contact with the herd, this was consistent with a diagnosis of chronic PJI. Based on the mNGS test results indicating a *C. burnetii*, which is sensitive to Doxycycline and Quinolones based on drug susceptibility testing, so oral Doxycycline (0.1 g po q12h) therapy for 7 days (Fig. 4). Further revision surgery was then needed, so treatment with Doxycycline (0.1 g ivgtt q12h) and Moxifloxacin (0.4 g ivgtt qd) was initiated for 25 days (Fig. 4), and the above 3 indexes were monitored in real time. Once the infection markers had stabilized, a tibial pad replacement surgery was performed on the knee joint. Postoperatively, the patient received Doxycycline (0.1 g ivgtt qd) and Moxifloxacin (0.4 g ivgtt qd) for 16 days (Fig. 4). A follow-up examination revealed an ESR was 17 mm/h, a CRP level was 0.94 mg/L, and the WBC count was 3.34 × 10⁹/L (Fig. 3). Symptoms of left knee pain and swelling showed marked improvement. No recurrence of left knee pain or swelling was observed during follow-up.

## 3. Discussion

The *C. burnetii* is a Gram-negative bacterium belonging to the Coxiellaceae family and the Legionellales order. It is a small, polymorphic rod (0.2–0.4 μm wide and 0.4–1.0 μm long) that occurs in 2 forms: the large cell variant (LCV) and the small cell variant (SCV). The LCV is the larger, less electron-dense intracellular form of the pathogen that is metabolically active. The spores of the LCV differentiate to produce the SCV, a resistant, spore-like form. The SCV is released when the cell lyses and can survive for long periods in the environment.^[8]^ It is recognized as an important zoonotic pathogen infecting a variety of domestic and wildlife species, including mammals, birds, and reptiles. However, cattle, sheep, and goats are considered the primary hosts of the pathogen. The bacterium can be transmitted by various routes, including respiratory, digestive, and direct contact with skin and mucous membranes.^[9]^ Additionally, studies indicate that *C. burnetii* aerosols can disperse up to 30 kilometers away.^[10]^ The microorganism lives and multiplies in the host’s monocytes, macrophages, and trophoblast cells. As it multiplies, it destroys host cells and continues to live in other cells.^[11]^ Potential consequences include endocarditis, vascular infections, bone and joint infections, lymphadenitis, and interstitial lung disease. In addition to conventional manifestations, atypical infections may present with neurological, ocular, and other related symptoms. One study reported that patients with chronic, limited *C. burnetii* infection have a 7.3% chance of developing bone and joint infections.^[12]^ In this study, the patient returned home shortly after undergoing left knee replacement surgery and proceeded to clean a cattle shed. Therefore, we infer that the patient contracted a *C. burnetii* infection through inhaling aerosols containing the pathogen or through skin contact with cattle excrement harboring the pathogen.

PJI is one of the most serious challenges for joint surgeons, with an incidence rate ranging from 2% to 2.4%.^[13]^ This greatly increases patient suffering. Most infections in total joint arthroplasty are associated with Gram-positive bacteria; the most common causative organisms are Staphylococcus aureus and S. epidermidis.^[14]^ Although traditional bacterial culture methods are the gold standard for diagnosing PJI, false-negative results sometimes occur. Due to the complexity of PJI, the diversity of pathogenic bacteria, and the intricate relationship between microbes and the host, some studies have shown that 50% of PJI patients after total knee arthroplasty (TKA) have negative bacterial cultures.^[15,16]^ The multiple polymerase chain reaction method can only identify a limited number of microorganisms. This method may miss uncommon pathogens and fail to detect fungal or mixed microbial infections. However, mNGS is widely used to diagnose and treat PJI. Studies have reported that mNGS is 94% sensitive and can effectively avoid false negatives, whereas traditional bacterial culture is only 70% sensitive.^[17]^ Furthermore, traditional testing methods often require 3 to 7 days to obtain results. Once an infection occurs, it progresses rapidly, which hinders early diagnosis and the application of antibiotics.^[18]^ This study employed an mNGS detection method capable of covering 25,000 pathogens and identifying pathogenic bacteria within 24 hours. After joint fluid aspiration, the Willingmed PIseq® high-throughput sequencing platform was used for analysis. Three data pipelines were employed: the platform’s proprietary database; the Chinese Pathogen Knowledge Base; and a dual-engine species identification algorithm. The analysis yielded 1308 highly specific sequences with excellent genomic coverage reaching 118% and a uniform distribution across the entire genome^[19]^ (supplementary material, Supplemental Digital Content, https://links.lww.com/MD/Q966). Additionally, a Q30 score of 94.19% and significant relative abundance of 4.57% were observed. mNGS identified a highly specific pathogen with concentrated signals, strongly suggesting that this pathogen is the primary cause of the infection. Provides valuable information for the early identification of pathogens and the guidance of antibiotic treatment. However, this study’s single-case design limits its ability to determine whether mNGS-based diagnostic and therapeutic strategies can universally improve the cure rate for PJI.

CRP and ESR are important indicators for evaluating inflammation and identifying postoperative infections. At the 2018 International Consensus Meeting, serum CRP, serum ESR, and elevated serum D-dimer were defined as secondary criteria in the revised International Consensus Meeting criteria.^[20]^ To improve the accuracy of diagnosing periprosthetic infections, combining ESR and serum CRP levels is recommended.^[21]^ The detection of elevated CRP and ESR levels can diagnose postoperative infection after TKA. Once the infection is controlled and the levels gradually return to normal, a second-stage revision surgery can be performed to remove the spacer prosthesis and implant a definitive prosthesis.^[22]^ Typically, CRP levels normalize within 2 weeks after TKA. PJI is considered if CRP levels remain above normal 1 month after surgery. Similarly, PJI is considered if ESR levels remain high 3 months after surgery.^[23]^ In this study, patients showed elevated CRP and ESR levels 9 months after surgery (Fig. 6). These 2 indexes gradually increased at the beginning of treatment, while there were no significant changes in WBC counts from the time of TKA to the effective control of PJI. With the combined application of antibiotics before and after surgery, the levels of CRP and ESR gradually decreased and stabilized and then decreased further to normal levels.

The clinical treatment of PJI mostly involves debridement and prosthesis preservation in a 1-stage or 2-stage revision.^[24–26]^ For superficial infections that have not yet reached the joint cavity, a conventional antibiotic regimen can be used, such as vancomycin combined with a cephalosporin.^[27]^ Then, sensitive antibiotics are used according to the results of the bacterial culture. Deep-level infections are often manifested by biofilm-protected bacterial adherence to the prosthesis,^[28]^ which is more drug-resistant. *C. burnetii* expresses multiple adhesins on its surface that recognize and bind to host cell membrane proteins on the prosthesis surface, thereby mediating the initial attachment of bacteria. At the same time, *C. burnetii* proliferates extensively, forming complex intracellular colonies that serve as “biofilm equivalents.”^[7]^ These structures are composed of lipopolysaccharide (LPS), proteins and DNA, and functionally resemble biofilms. They provide a microenvironment that is sheltered from extracellular antibiotics and immune factors.^[29,30]^ Furthermore, studies indicate that *C. burnetii* lipopolysaccharide modulates monocyte phagocytosis and bacterial survival, likely contributing to evasion of host immune responses. This occurs through the suppression of dendritic cell maturation, as well as the participation in atypical M2 activation of human macrophages.^[7]^ Conservative treatment relying on antibiotics alone is no longer able to control the infection; therefore, surgical intervention is often required. For knee PJI, 1-stage or 2-stage revision can be chosen. One-stage revision involves the 1-time removal of the infected prosthesis and placement of a new prosthesis after thorough debridement. The new prosthesis is fixed with long-acting antibiotic bone cement. Treatment with sensitive antibiotics is continued for more than 6 weeks postoperatively to control the infection. Two-stage revision is often considered the gold standard for treating postoperative PJI after TKA,^[31,32]^ which requires 2 surgical procedures: one to remove the infected prosthesis and leave the area open with antibiotic-impregnated bone cement and a second procedure to implant a new prosthesis. One study compared the success rates of 1-stage and 2-stage revisions for treating PJI. The study found that the early success rate of 1-stage revision (94.2%) was higher than that of 2-stage revision (84.0%). However, the intermediate-term and long-term success rates were similar for both methods.^[33]^ For this study, patients underwent primary reconstructive surgery only after infection indicators were effectively controlled. There was no sign of reinfection at the 1-year postoperative follow-up. For antibiotic selection, Tetracyclines (Doxycycline, Minocycline, etc) and Quinolones (Moxifloxacin, Ciprofloxacin, etc) were used in combination based on the mNGS test results. In this study, the patient’s knee infection was effectively controlled through surgery and the combined use of antibiotics.

## 4. Conclusions

In summary, mNGS testing allows for the rapid and accurate identification of the bacteria causing PJIs after TKA. This testing method effectively guides antibiotic use and facilitates timely surgical intervention.

## Author contributions

**Data curation:** Yunxin Sun, Qiang Shang.

**Investigation:** Jie Mei.

**Supervision:** Yubao Yang, Jinqing Kan, Xiaobing Chen.

**Visualization:** Peilei Sun.

**Writing – original draft:** Hao Mei.

**Writing – review & editing:** Hao Mei, Luchun Sun.

## Supplementary Material


