# Supplementary material for: Antibiotics guided by metagenomic next-generation sequencing to control infection after total knee arthroplasty: A case report and literature review
Source: Medicine (Baltimore). 2025 Dec 19;104(51):e46734. doi: 10.1097/MD.0000000000046734 (PMC12727269; doi:10.1097/MD.0000000000046734)

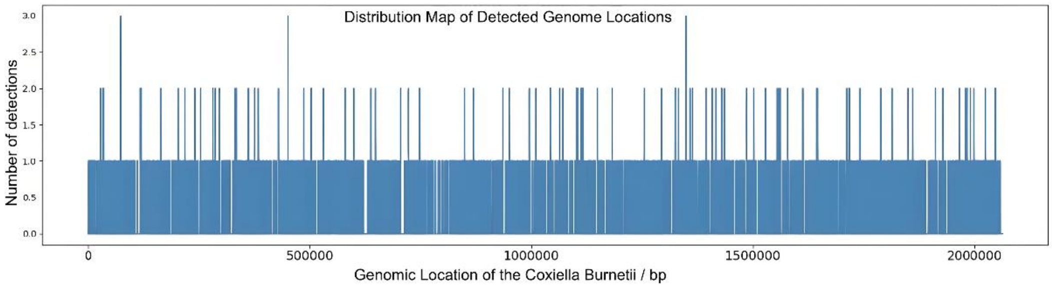

Supplementary Material: Reads obtained from mNGS are located in the position of Coxiella burnetii genome.

一、样本与诊断信息

受检者基本信息

姓名：[redacted]

性别：男

年龄：74

电话：—

住院号：[redacted]

床号：—

受检者临床诊断信息

临床症状：—

临床诊断：—

血常规：

WBC（10^9/L）	淋巴细胞（%）	中性粒细胞（%）	CRP（mg/L）	PCT（ng/ml）
—	—	—	—	—

其他结果：—

重点关注：病毒、细菌、真菌、寄生虫、结核、支/衣原体、耐药基因

近期用药：—

样本信息

条码号：[redacted]

样本编号：[redacted]

样本类型：关节积液

样本体积：5ml

样本采集时间：2023-10-09 14:33:00

样本接收时间：2023-10-10 10:18:30

样本质量：未见异常

送检医生：[redacted]

送检科室：骨科

送检单位：[redacted]

检测项目：PIseq®DNA病原检测产品

二、检测结果

检出指标	序列数(RPTM)[1]	阳性参考范围	定性结果[2]
贝氏柯克斯体	1308	≥1	细菌阳性

[1]RPTM，每一千万条测得序列包含的阳性序列条数(Reads per ten million)。

[2]定性结果同时也参考了内置的置信度指数计算模型值，用于描述检出物种的技术可靠度，主要包括阳性、疑似、人体微生态等结果。

本报告内容仅供专业的研究人员及临床医生参考，不作为临床确诊的唯一依据。

三、附表 - 详细检测信息

1、样本中物种核酸丰度比例

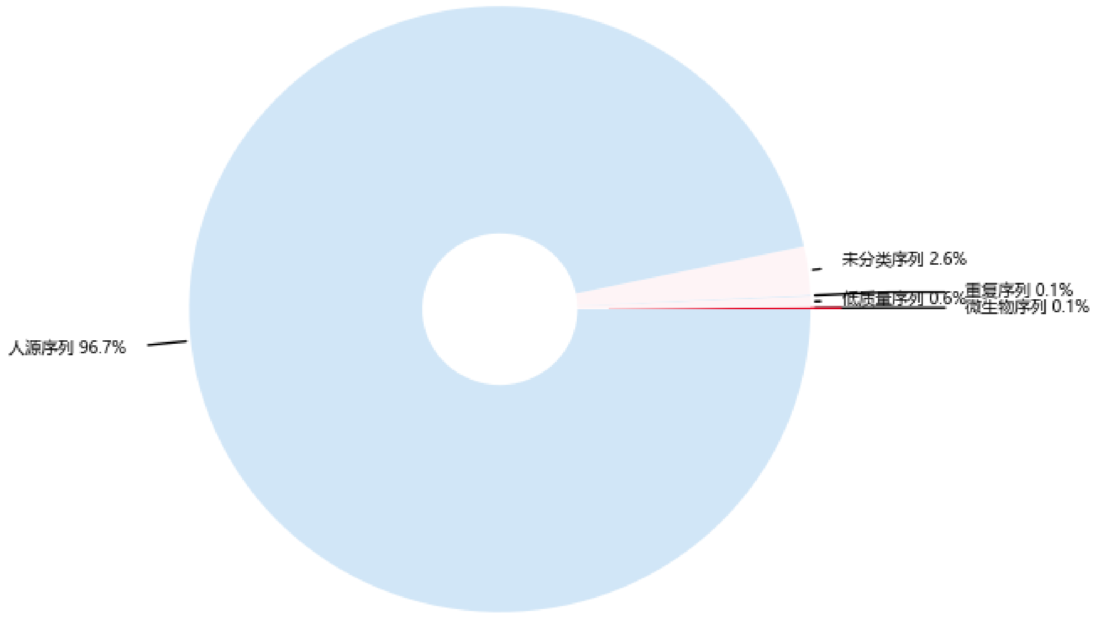

2、病毒筛查结果

序号	属信息		种信息			
	属名	序列数	种名	序列数	覆盖度	相对丰度
	未检出					

3、细菌筛查结果

序号	属信息		种信息			
	属名	序列数	种名	序列数	覆盖度	相对丰度

1	Coxiella<br>（柯克斯体属）	1308	Coxiella_burnetii<br>（贝氏柯克斯体）	1308	94195 bp<br>4.57%	11.8%

贝氏柯克斯体是一种专性细胞内寄生的革兰阴性微小细菌，为柯克斯体属细菌，在自然界广泛分布。该菌是一类重要的人兽共患细胞内寄生菌，主要通过呼吸道吸入病原体被感染，一般农民、兽医、屠宰场工人以及实验室工作人员易感本菌，可导致不明发热—俗称Q热，或伴有肺炎、心内膜炎、肝炎、脊髓炎等。

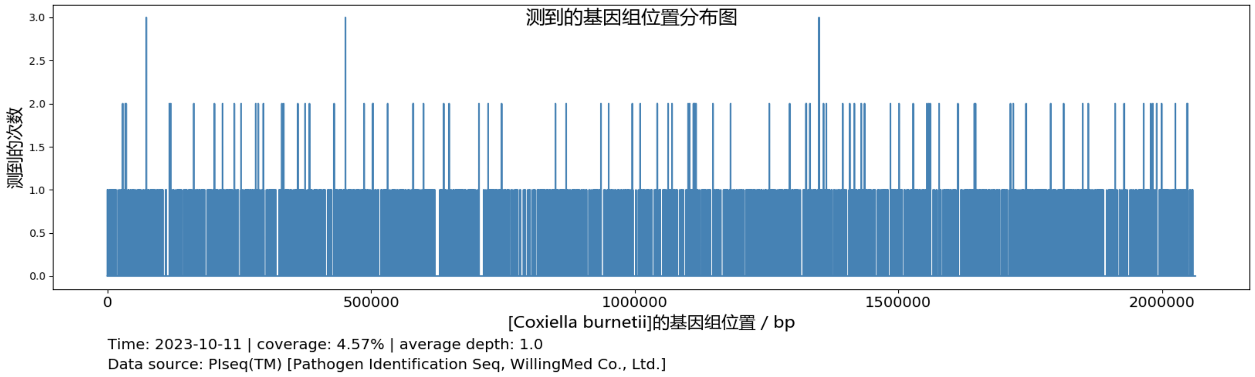

4、真菌筛查结果

序号	属信息		种信息			
	属名	序列数	种名	序列数	覆盖度	相对丰度
	未检出					

5、寄生虫筛查结果

序号	属信息		种信息			
	属名	序列数	种名	序列数	覆盖度	相对丰度
	未检出					

6、耐药基因筛查结果

序号	检测出耐药基因	抗生素类别	可能对应物种
	未检出		

7、毒力基因筛查结果

序号	毒力因子	基因名称	功能	可能对应物种

1	Dot/Icm_T4SS	icmE[4]	该因子能够促进许多细菌效应蛋白跨细菌膜和液泡膜转运进入宿主细胞质，在该细胞质中，效应蛋白通过调节自身的特异性活动来调控宿主细胞的各种过程。	Q热病原体/伯氏考克斯氏体

8、检出常见人体微生态细菌列表

序号	属信息		种信息				注释
	属名	序列数	种名	序列数	覆盖度	相对丰度	
	未检出						

四、质量控制信息

指标	结果
阳性对照	合格
阴性对照	合格
内对照	合格
GC%	42%
Q30	94.190%

测序质量分布图：

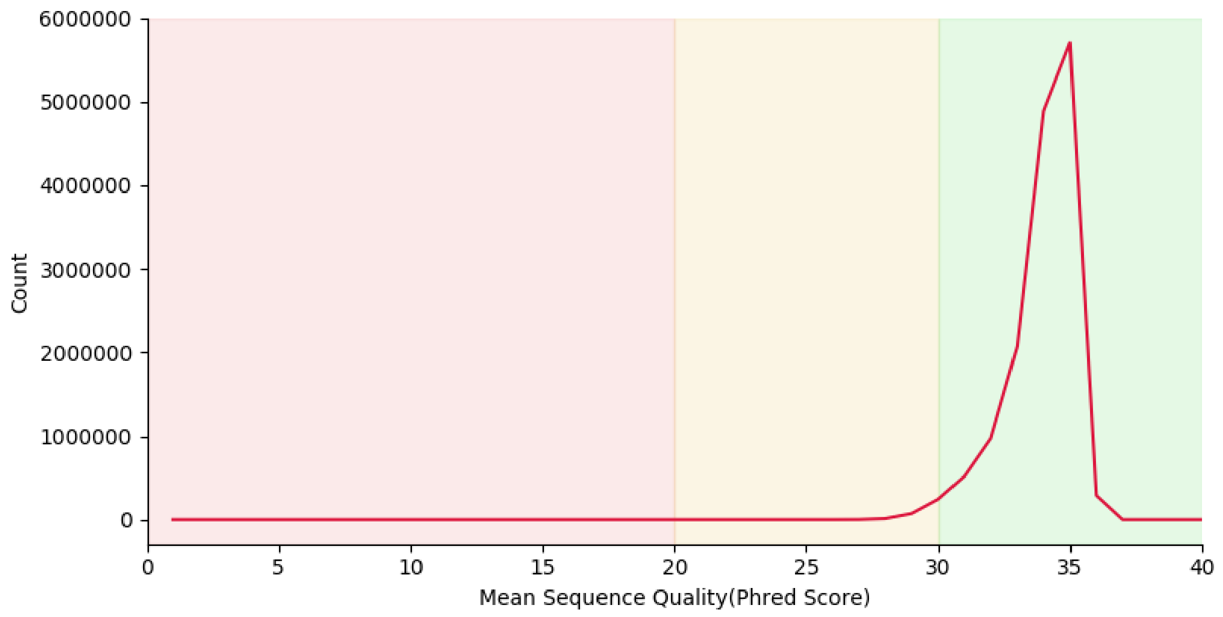

注：以上图表说明此次检测测序数据质量合格，结果可信。

五、检验原理和术语、名词说明

PIseq®（Pathogen Identification Sequencing）是微岩医学基于宏基因组学二代测序技术（mNGS，metagenomics next-generation sequencing）开发的病原微生物检测技术，该技术无需预判感染微生物，直接对临床样本中的遗传物质（核酸）进行全面无偏的检测。PIseq®技术可覆盖25000多种病原体，包括11836种细菌、11021种病毒、1872种真菌、421种寄生虫、153种分枝杆菌、118种支原体/衣原体、以及105种立克次体。本检测通过高效病原富集核酸提取技术，结合微岩自建的微生物基因标签序列数据库及中国流行病原知识库，采用独有的双引擎物种鉴定算法，全面扫描样本中存在的病原微生物、耐药基因和毒性元件等信息，并提供大信息量的检测报告及报告解读， 在不明感染、疑难危重及免疫缺陷感染患者中有很高的临床应用价值。

- 检测流程

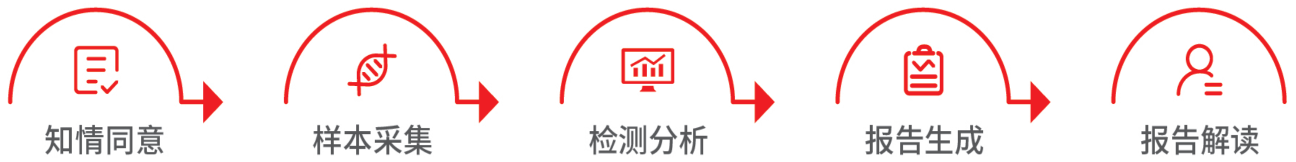

- 检测范围

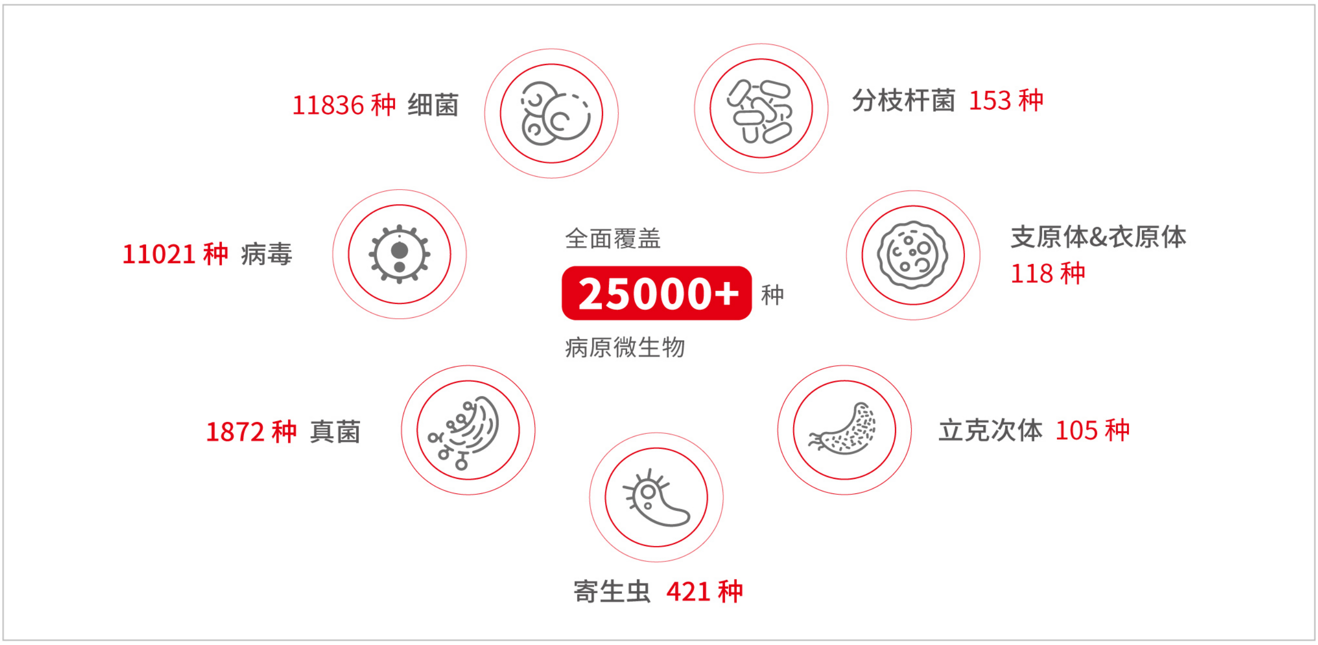

Supplement: Supplementary file 1 [file medi-104-e46734-s001.pdf]
